# Supplementary material for: Mating system manipulation and the evolution of sex-biased gene expression in Drosophila
Source: Nat Commun. 2017 Dec 12;8:2072. doi: 10.1038/s41467-017-02232-6 (PMC5727229; doi:10.1038/s41467-017-02232-6)
Supplement: Supplementary file 1 — Supplementary Information [file 41467_2017_2232_MOESM1_ESM.pdf]

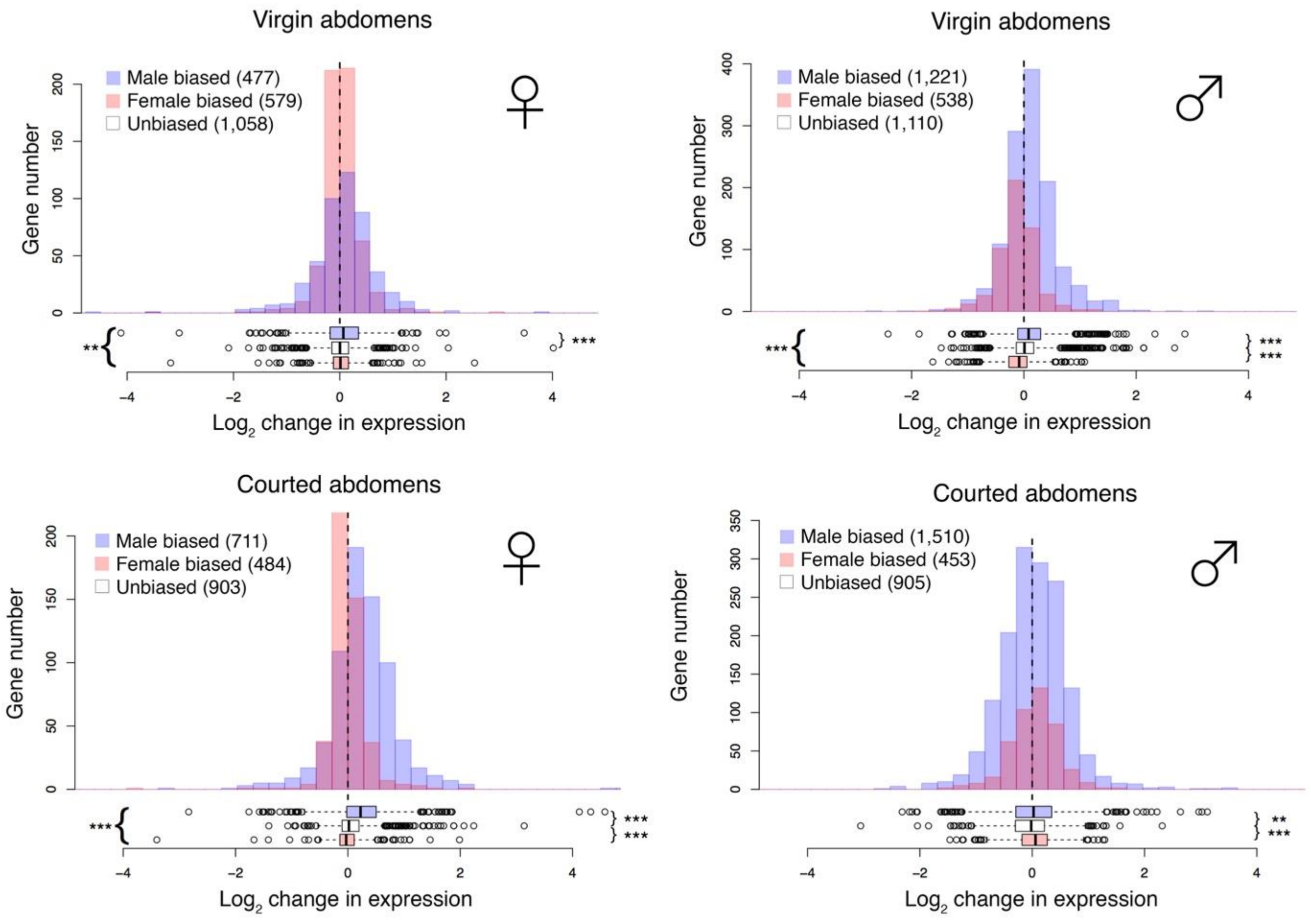

Supplementary Figure 1. Gene expression changes following experimental manipulation of mating system, after removing testes- and ovary-biased genes. Positive values on the x axis correspond to higher expression under Monogamy, negative values to high expression under increased Polyandry. Colours indicate male- (blue), female- (pink) and un- (white) biased genes in virgin abdomens (top) and courted abdomens (bottom). For clarity, unbiased genes are omitted from the histogram. The significance level of Mann-Whitney rank tests on the average level of sex-biased expression is indicated by asterisks (***<0.001, **<0.01). Asterisks to the right of the box plots summarise comparisons of male- and female- biased genes with unbiased genes, and those to the left between male- and female- biased genes.

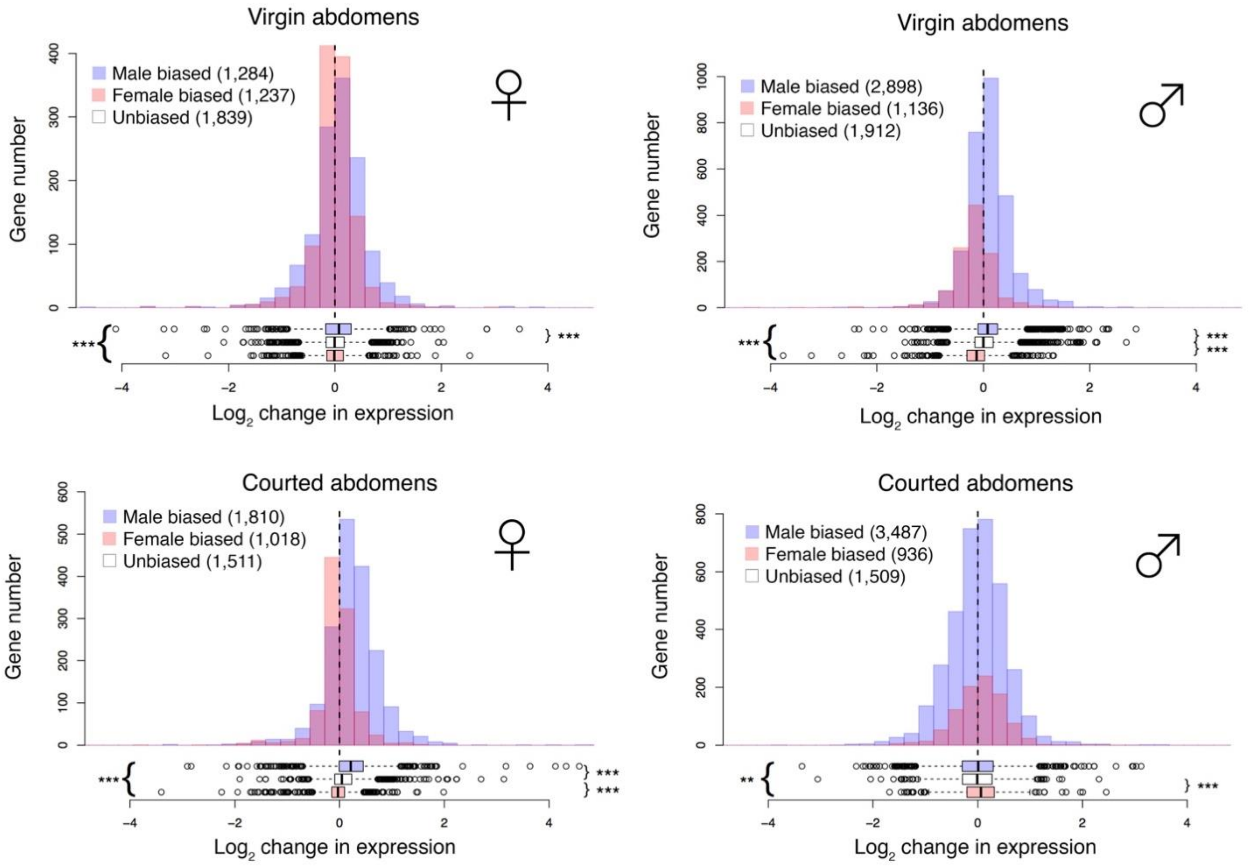

Supplementary Figure 2. Gene expression changes following experimental manipulation of mating system, after removing sex-specific genes. Positive values on the x axis correspond to high expression under Monogamy, negative values to high expression under increased Polyandry. Colours indicate male- (blue), female- (pink) and un- white) biased genes in virgin abdomens (top) and courted abdomens (bottom). For clarity, unbiased genes are omitted from the histogram. The significance level of Mann-Whitney rank tests on the average level of sex-biased expression is indicated by asterisks (***<0.001, **<0.01). Asterisks to the right of the box plots summarise comparisons of male- and female- biased genes with unbiased genes, and those to the left between male- and female- biased genes.

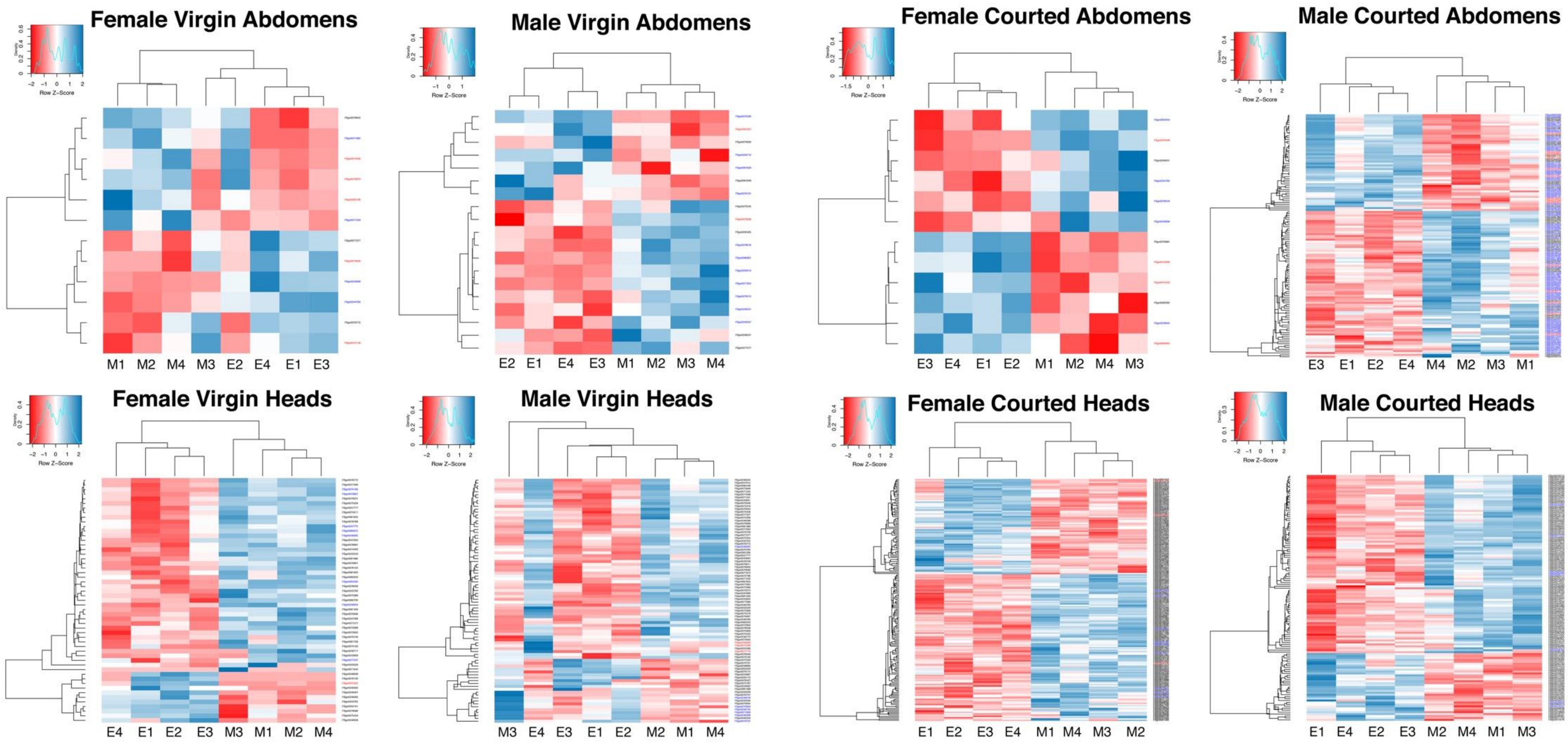

Supplementary Figure 3. Heatmaps of the expression level of DE genes in (a) the 4 virgin and (b) the 4 courted contrasts investigating response to experimental sexual manipulation. The clustering is based on average expression. Columns represent libraries, rows represent genes. Gene names are colour coded based on their sex bias in that tissue and sex (red - female-biased, blue - male biased). More information on each gene is available in Supplementary Data 1.

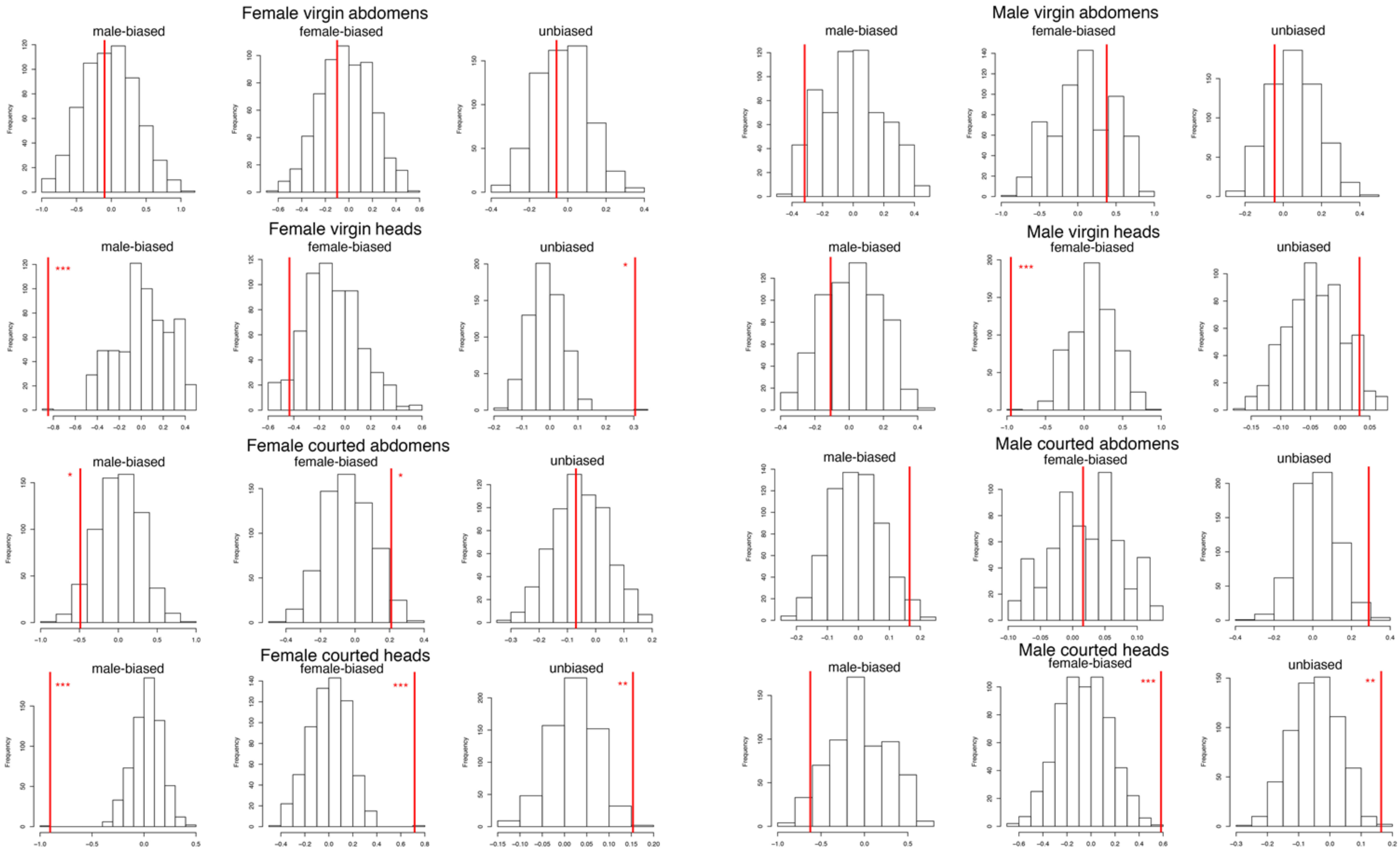

Supplementary Figure 4. Results of the standard randomisation approach. Histograms summarise all 630 possible iterations of groups of 4 replicates, each with 2 treatment types. Red bars indicate the observed mean stdLogFC. Positive values in the x axis indicate higher expression under Polygamy. Stars indicate the proportion of iterations with a more extreme value than the observed (*** < 0.001, ** < 0.01, * < 0.05).

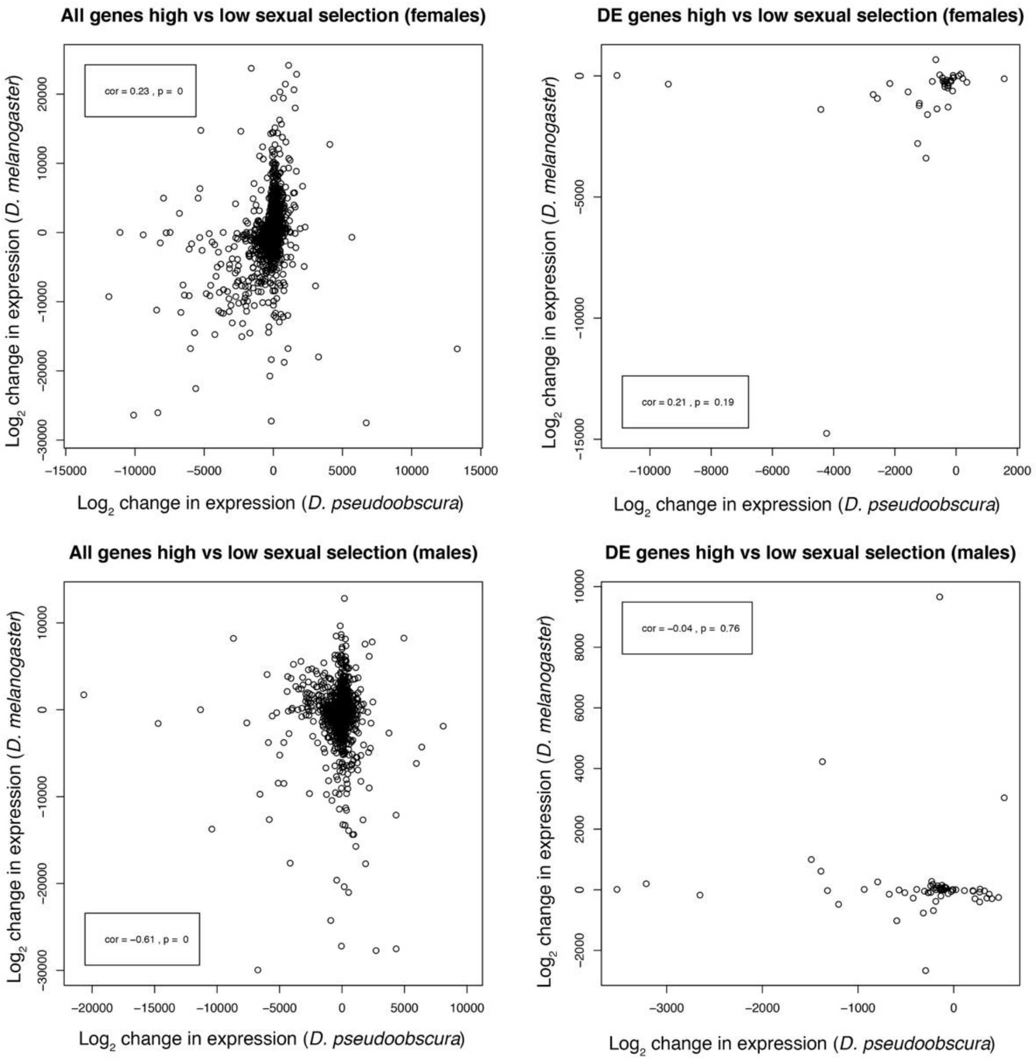

Supplementary Figure 5. **Correlation plots of expression level changes in response to sexual selection of genes with homologues in *D. melanogaster,* in virgin heads in Hollis *et al.*[1] and this study.** Left is all genes (after removal of the top & bottom 0.5% to minimise effects due to outliers) and right is involving only genes found to be significantly differentially expressed in this study. Correlation coefficients and p values are given in insets.

Supplementary Table 1. The mean and 95% confidence intervals of fold changes in expression level of female-, male- and un-biased genes under monogamy, as determined from 10,000 bootstraps from the raw data (original gene numbers = N). Results are presented separately for each tissue type examined, in terms of feminisation/masculinisation under monogamy. An ANOVA of the original data is also presented, and indicates that female-, male- and un-biased genes significantly differed in response to the treatment in all cases.

Contrast	Bias type	N	MeanFC	Stan. Error.	Lower95% CI	Upper95% CI	>/< 0?	Direction under monogamy	ANOVA				
Virgin female abdomens	female	4219	0.0031	0.0042	-0.0054	0.0112	NS		**Source**	d.f.	s.s.	v.r.	F pr.
	male	1832	0.0244	0.0123	0.0008	0.0482	~>0	~Masculinised	**Bias type**	2	0.61	2.79	0.062
	unbiased	3296	0.0039	0.0055	-0.0079	0.0139	NS		**Residual**	9320	1129.9		
Virgin male abdomens	female	4092	-0.1911	0.0054	-0.2017	-0.1807	<<0	Masculinised	**Source**	d.f.	s.s.	v.r.	F pr.
	male	4631	0.0819	0.0052	0.0718	0.0922	>0	Masculinised	**Bias type**	2	171.7	730.6	<.001
	unbiased	3372	0.0174	0.0055	0.0064	0.0281	>0		**Residual**	12092	1421.1		
Virgin female heads	female	220	0.0526	0.0616	-0.0722	0.1677	NS		**Source**	d.f.	s.s.	v.r.	F pr.
	male	73	0.681	0.125	0.444	0.936	>>0	Masculinised	**Bias type**	2	31.2	46.26	<.001
	unbiased	10587	0.0274	0.0055	0.0163	0.0381	~>0		**Residual**	10877	3677.2		
Virgin male heads	female	203	0.4090	0.0841	0.2572	0.5868	>>0	Feminised	**Source**	d.f.	s.s.	v.r.	F pr.
	male	96	-0.336	0.158	-0.666	-0.045	<0	Feminised	**Bias type**	2	42.6	60.4	<.001
	unbiased	10550	0.0243	0.0055	0.0136	0.0350	>0		**Residual**	10846	3822.6		
Courted female abdomens	female	3780	-0.0418	0.0042	-0.0502	-0.0340	<0	Masculinised	**Source**	d.f.	s.s.	v.r.	F pr.
	male	2468	0.185	0.0296	0.1661	0.2041	>>0	Masculinised	**Bias type**	2	76.5	315.6	<.001
	unbiased	3075	0.0460	0.0056	0.0351	0.0570	>0		**Residual**	9320	1129.9		
Courted male abdomens	female	3674	0.0399	0.0060	0.0280	0.0516	>0	Feminised	**Source**	d.f.	s.s.	v.r.	F pr.
	male	5342	-0.0197	0.0064	-0.0324	-0.0071	<0	Feminised	**Bias type**	2	12.6	35.1	<.001
	unbiased	3073	-0.0418	0.0073	-0.0561	-0.0274	<0		**Residual**	12086	2176.7		
Courted female heads	female	105	-0.3202	0.0747	-0.4635	-0.1690	<<0	Masculinised	**Source**	d.f.	s.s.	v.r.	F pr.
	male	197	0.3942	0.0377	0.3212	0.4703	>>0	Masculinised	**Bias type**	2	39.4	51.8	<.001
	unbiased	10693	0.0217	0.0059	0.0097	0.0335	>0		**Residual**	10992	4186.2		
Courted male heads	female	93	-0.2313	0.0572	-0.3480	-0.1260	<<0	Masculinised	**Source**	d.f.	s.s.	v.r.	F pr.
	male	216	0.2474	0.0468	0.1562	0.3383	>>0	Masculinised	**Bias type**	2	21.1	30.4	<.001
	unbiased	10608	-0.0405	0.0057	-0.0516	-0.0293	<0		**Residual**	10916	3787.8		

Supplementary References

1. Hollis, B., Houle, D., Yan, Z., Kawecki, T. J. & Keller, L. Evolution under monogamy feminizes gene expression in *Drosophila melanogaster*. *Nature Communications* **5**, (2014).
