# Supplementary material for: Mating system manipulation and the evolution of sex-biased gene expression in Drosophila
Source: Nat Commun. 2017 Dec 12;8:2072. doi: 10.1038/s41467-017-02232-6 (PMC5727229; doi:10.1038/s41467-017-02232-6)
Supplement: Supplementary file 3 — Description of Additional Supplementary Files [file 41467_2017_2232_MOESM3_ESM.pdf]

File Name: Supplementary Data 1

Description: GO analysis of the differentially expressed (FDR = 10%) genes in each of the 8 contrasts (different combinations of sex, courtship status and tissue) between the lines evolved under Monogamy and Polyandry. Each tab describes a contrast, and within a tab there is a summary of all DE genes, those upregulated under Monogamy and those upregulated under Polyandry. The columns indicate the major GO category (BP: Biological process, CC: Cellular component, MF: Molecular function), the number of all genes and number of DE genes included in the contrast that is annotated with the GO term, and the significance level of either a simple Fisher's exact test on GO counts or a Fisher's exact test after accounting for GO topology (topGO Fisher column). The latter was used to filter the table to only include terms with p values < 10%.

File Name: Supplementary Data 2

Description: GO analysis of the differentially expressed (FDR = 10%) genes in each of the 4 contrasts (made up of combinations of sex and tissue) between the Virgin and Courted flies of Baseline. Each tab describes a contrast, and within a tab there is a summary of all DE genes, those upregulated in virgins and those upregulated in courted flies. The columns indicate the major GO category (BP: Biological process, CC: Cellular component, MF: Molecular function), the number of all genes and number of DE genes included in the contrast that is annotated with the GO term, and the significance level of either a simple Fisher's exact test on GO counts or a Fisher's exact test after accounting for GO topology (topGO Fisher column). The latter was used to filter the table to only include terms with p values < 10%.
